# Pneumonia in an elderly Tibetan male caused by *Shewanella* algae: A case report

**DOI:** 10.1097/MD.0000000000039197

**Published:** 2024-08-09

**Authors:** Xiao-Hong Fu, Huan-Xiang Feng, Qian Huang, Wei Gou

**Affiliations:** a Department of Medical Laboratory, Chengdu Seventh People’s Hospital, Chengdu, China; b Department of Neurology, Chengdu Seventh People’s Hospital, Chengdu, China.

**Keywords:** case report, pulmonary infection, *Shewanella* algae, Tibetan

## Abstract

**Rationale:**

*Shewanella* algae are Gram-negative bacteria that are widely found in aquatic habitats and rarely cause lung infections in inland areas.

**Patient concerns:**

Cough with light-yellow phlegm for 2 weeks.

**Diagnoses:**

The final diagnosis was bacterial pneumonia.

**Interventions:**

The patient was treated with ceftazidime (2 g, every 12 h) for 1 week.

**Outcomes:**

The patient’s lung infection improved and he was discharged.

**Lessons:**

This case highlights a rare occurrence of lung infection caused by *Shewanella* algae in elderly Tibetan men residing in non-marine environments.

**Key points**

- *Shewanella* algae are opportunistic pathogens with definite life histories that usually cause diseases. *Shewanella* algae can not only cause skin and soft tissue infections and otitis media, but also cause lung infections, especially in people with underlying diseases. Although the symptoms of lung infection did not cause serious adverse consequences, it reminds us that their scope of influence is expanding and needs to be considered.

## 1. Introduction

*Shewanella* algae are widely distributed, non-fermenting, Gram-negative bacteria found in aquatic habitats.^[1]^ This infection is typically observed in individuals with compromised immune function following trauma from open water.^[2]^ It is known to cause various types of infections, including skin and soft tissue infections, otitis media, vertebral discitis, bloodstream infections, and abdominal infections.^[3,4]^ However, recent global reports have indicated that *Shewanella* infections are not limited to tropical and temperate regions during summer, nor are they exclusive to individuals in contact with open water. Cases of *Shewanella* infections occurring in non-tropical regions and individuals not exposed to open water have been documented.^[5,6]^ This case report describes the rare occurrence of pneumonia caused by *Shewanella* algae in an individual who consumed raw shrimp in inland China. *Shewanella* are uncommon opportunistic pathogens that cause pneumonia. This case highlights the significance of *Shewanella* as an uncommon opportunistic pathogen capable of causing pneumonia and serves as a crucial reminder for physicians to be vigilant about the potential of *Shewanella* algae to cause infections at various anatomical sites.

## 2. Case presentation

### 2.1. Chief complaints

A 68-year-old Tibetan male presented to our neurology clinic complaining of persistent cough with light-yellow phlegm for 2 weeks.

### 2.2. History of present illness

Symptoms started 2 weeks before the persistent cough with light-yellow phlegm, without any apparent cause. He measured his body temperature at home and recorded it as 37.3 °C, along with experiencing chills. He took unspecified medications independently, but the symptoms did not improve significantly. In addition, the patient reported fatigue and shortness of breath after physical activities. He denied experiencing chest tightness, palpitations, nausea, or vomiting. Owing to these symptoms and a history of related neurological disorders, the patient sought further treatment in the neurology department. Sequential Organ Failure Assessment score: 3; confusion, urea, respiratory rate, blood pressure, age >65 score: 2.

### 2.3. History of past illness

Medical history included occasional lower back pain, which had worsened bilaterally over the past 2 weeks without prior diagnosis or treatment. The patient had a history of cerebral infarction, epilepsy, stage 4 chronic kidney disease, renal anemia, gout, stable angina pectoris, coronary atherosclerotic heart disease, coronary muscle bridge, grade 2 high-risk primary hypertension, type 2 diabetes, atrial fibrillation, and cholecystectomy.

### 2.4. Personal and family history

Nothing in particular.

### 2.5. Physical examination

On examination, the patient had: body temperature, 36.5 °C; pulse rate, 80 beats/min; respiratory rate, 20 breaths/min; blood pressure, 138/84 mm Hg; oxygen saturation level, 98% (in an unoxygenated state). General examination revealed normal development, good nutrition, consciousness, autonomous posture, acute illness, physical examination cooperation, entering the ward, and normal gait. Respiratory sounds in both lungs were rough; respiratory sounds in the right lung were low, audible, and wheezing, heart rhythm was absolutely irregular, first heart sound varied in strength, pulse rate was less than heart rate, heart sound varied in strength. No split heart, heart murmur, pericardial friction, and additional heart sound was heard. Direct percussion caused pain near the posterior superior iliac spine on both sides.

### 2.6. Laboratory examinations

**Figure 1.**
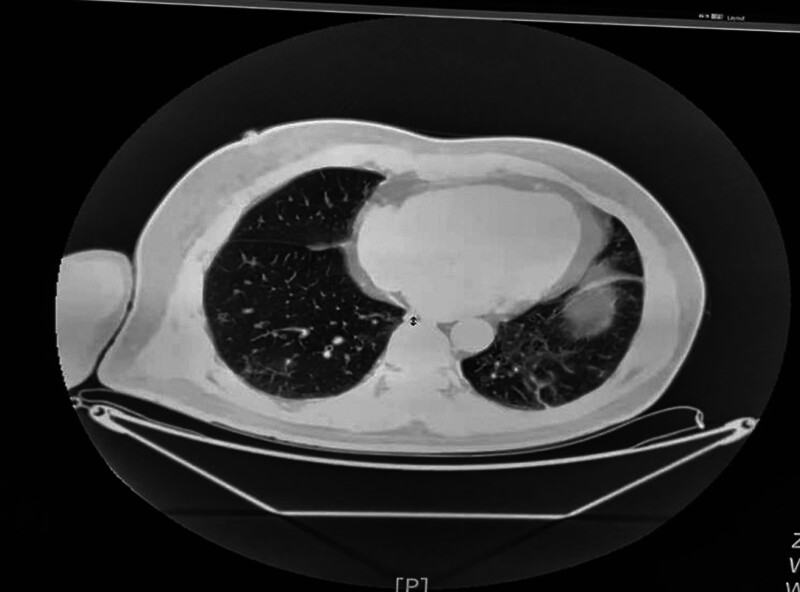
Pure culture of blood agar culture-medium.

Blood test results revealed the following: white blood cell count, 10,030/μL and procalcitonin 0.067 ng/mL. A screening test for the respiratory 2019-novel coronavirus using throat swabs yielded negative results. Liver function tests revealed levels of albumin, 37.5 g/L (normal range, 40–55 g/L) and adenosine deaminase, 23.9 U/L (normal range, 4–18 U/L), whereas renal function tests revealed levels of urea, 18.3 mmol/L (normal range, 3.6–9.5 mmol/L) and creatinine, 264.0 μmol/L (normal range, 57–111 μmol/L) and glomerular filtration rate, 21 mL/min (normal range, 80–120 mL/min); no other abnormalities were found. The lower respiratory tract [epithelial cells < 10/lower power field and leukocytes > 25/ lower power field] was collected and cultured, and a Gram-negative bacterium that tested positive for oxidase was isolated after 24 hours of incubation. The bacterium was further identified as *Shewanella* algae using a BioMerieux VITEK 2 Compact, matrix-assisted laser desorption/ionization time of flight mass spectrometry (Autof ms1000), and 16S ribosomal RNA gene sequence analysis (Fig. 1). The drug sensitivity was detected by broth microdilution method recommended by Clinical and Laboratory Standards Institute, and the drug sensitivity was judged according to Clinical and Laboratory Standards Institute relevant standards. Antibiotic susceptibility results are presented in Table 1.

**Table 1:** Results of antibiotic susceptibility testing against isolates in the study.

Antibiotics	MIC (μg/mL)	Sensitivity
Piperacillin	≤16	S
Ceftazidime	≤4	S
Cefepime	≤8	S
Levofloxacin	≤0.12	S
Meropenem	≤1	S
Aztreonam	≥32	R
Gentamicin	≤4	S
Minocycline	≤4	S

MIC = Minimum Inhibitry Concentration, R = resistance, S = susceptible.

### 2.7. Imaging examinations

**Figure 2.**
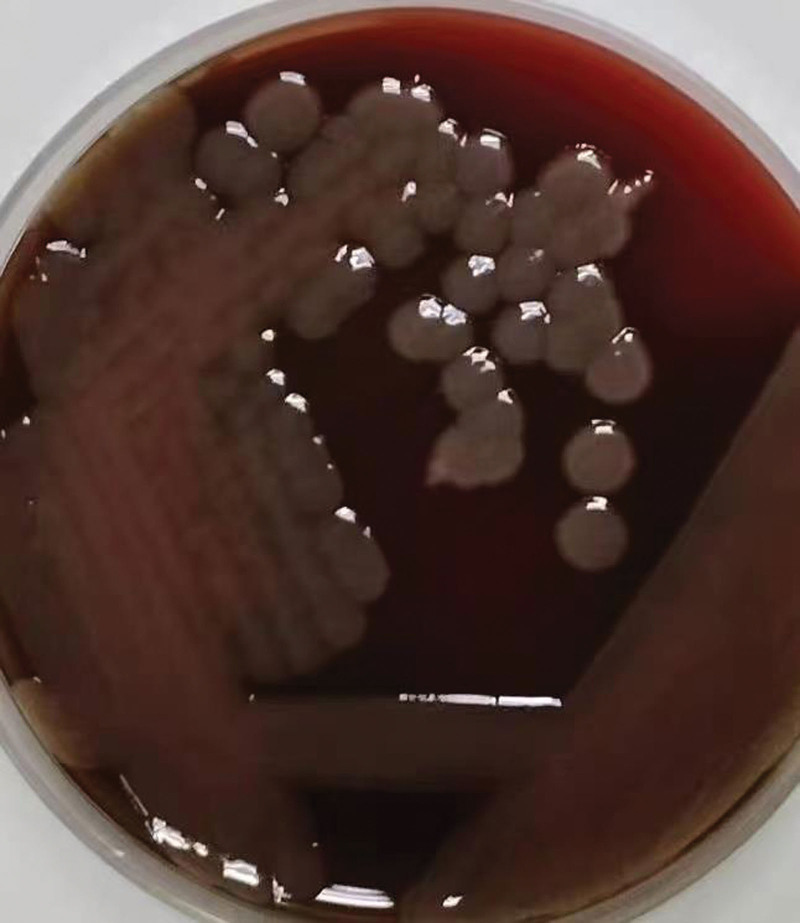
Chest computed tomography results of the patient after admission.

Computed tomography showed scattered lesions (Fig. 2).

## 3. Final diagnosis

The final diagnosis was bacterial pneumonia, and other diagnoses included grade 2 hypertension (very high risk), type 2 diabetes, secondary epilepsy, coronary atherosclerotic heart disease, atrial fibrillation, chronic kidney disease stage 4, renal anemia, and lacunar infarction.

## 4. Treatment

The patient was treated with ceftazidime (2 g, every 12 h) for 1 week, which improved the pneumonia symptoms. The patient was then discharged.

## 5. Outcome and follow-up

In June, the patient reported being in a good condition. In July, the patient returned to Lhasa, which is at an altitude of approximately 3000 m, and did not experience any respiratory symptoms.

## 6. Discussion

*Shewanella* algae is primarily known as an opportunistic pathogen that commonly causes skin and soft tissue infections, followed by bloodstream and biliary tract infections.^[5,6]^ Other types of infections are rarely reported, and most cases occur in coastal areas or in individuals exposed to seawater and seafood.^[7]^ Studies have identified several risk factors associated with *Shewanella* algae infection, including liver and bile diseases, chronic kidney disease, diabetes, malignant tumors, immune deficiency, chronic lower limb ulcers, and male gender, among others.^[6,8–10]^

After obtaining the identification results, the patient’s medical history was reviewed, and it was discovered that the patient had a history of raw shrimp consumption prior to disease onset. This led to speculation that this was the most likely route of infection in this case, which aligns with the findings of some studies.^[11]^ However, in most cases where patients have a history of consuming raw shrimp, biliary tract infections are the most common, with occasional cases of gastroenteritis being reported.^[12]^ Respiratory infections caused by *Shewanella* are rare.

One study analyzed 273 strains of *Shewanella* spp. (96 strains of *Shewanella* algae) published in PubMed between January 1978 and June 2022.^[6]^ It included 19 strains of *Shewanella* spp. in China, which caused chest infections in 12 (4.4%) cases. Another report showed that the proportion of respiratory infections caused by *Shewanella* spp. was 13%.^[13]^ Another study found that only 6 out of 29 isolated *Shewanella* spp. infection strains were *Shewanella* algae.^[14]^ Therefore, it can be inferred that pneumonia caused by *Shewanella* algae is rare.

A study in Hong Kong^[8]^ showed that 1 of every 6 cases of marine exposure was pneumonia. Other pathogens were detected at the same time, and it was determined that the possible pathogen was *Shewanella* spp. However, whether the pneumonia was caused by *Shewanella* algae was unclear. Therefore, reports of pneumonia caused by *Shewanella* algae are rare, particularly in China.

This case report describes pneumonia in an elderly Tibetan male with underlying diseases in inland China. Although the patient had multiple complex underlying diseases, the prognosis of pneumonia was good. *Shewanella* algae are often sensitive to third-generation cephalosporins, aminoglycosides, and quinolone antibiotics, but resistant to colistin and sulfonamides.^[1]^ In this case, the patient was empirically treated with the third-generation cephalosporin ceftazidime before the pathogen was clearly identified, and the patient’s condition improved after 1 week of use.

Notably, climate change may be associated with an increase in the number of *Shewanella*. The temperature of marine ecosystems can promote the growth and expand the reach of these pathogenic microbes in subtropical humid climates. Additionally, economic development has led to the expansion of fresh marine biological transport, which may contribute to the expansion of marine pathogenic microorganisms.

## 7. Conclusion

This report describes a unique case of pneumonia caused by *Shewanella* algae in inland China, with a history of raw shrimp consumption and underlying diseases. *Shewanella* algae are causing an increasing incidence of human infections in inland areas; however, pneumonia is rare. This case suggests that *Shewanella* algae cause more infections and increase the possibility of more site of infections with the expansion of marine biotransport. In terms of clinical diagnosis and treatment, this case can improve the understanding, diagnosis, and treatment abilities of physicians dealing with *Shewanella* algae infections in inland populations.

## Acknowledgments

We thank the patient and his family for provision of clinical data.

## Author contributions

**Conceptualization:** Xiao-Hong Fu, Wei Gou.

**Data curation:** Xiao-Hong Fu, Huan-Xiang Feng.

**Formal analysis:** Xiao-Hong Fu.

**Funding acquisition:** Xiao-Hong Fu.

**Investigation:** Xiao-Hong Fu.

**Methodology:** Xiao-Hong Fu.

**Project administration:** Xiao-Hong Fu.

**Resources:** Xiao-Hong Fu, Qian Huang, Wei Gou.

**Software:** Xiao-Hong Fu.

**Supervision:** Xiao-Hong Fu.

**Validation:** Xiao-Hong Fu.

**Visualization:** Xiao-Hong Fu.

**Writing – original draft:** Xiao-Hong Fu.

**Writing – review & editing:** Xiao-Hong Fu.
